# Supplementary material for: AI-based approach for transcribing and classifying unstructured emergency call data: A methodological proposal
Source: PLOS Digit Health. 2023 Dec 6;2(12):e0000406. doi: 10.1371/journal.pdig.0000406 (PMC10699611; doi:10.1371/journal.pdig.0000406)
Supplement: Attachment — Submitted filename: Response to Reviewers.pdf [file pdig.0000406.s001.pdf]

Response to Reviewers

Mengyu Wang, Ph.D.
Academic Editor
PLOS Digital Health

Submission Date: 07/14/2023

Dear Professor Wang,

Thank you for your careful review of our submission entitled "AI-based approach for transcribing and classifying unstructured emergency call data: A methodological proposal". We also appreciate the time and effort you and each of the reviewers have dedicated to providing insightful feedback on ways to strengthen our paper. Therefore, it is with great pleasure that we resubmit our article for further consideration. We have incorporated changes that reflect the detailed suggestions provided. We also hope that our edits and the responses we provide below satisfactorily address all the issues and concerns you and the reviewers have noted.

To facilitate your review of our revisions, the following is a point-by-point response to the questions and comments delivered in your letter. We have also included a marked-up copy (pdf) of the manuscript where changes are highlighted in red.

EDITOR SUGGESTIONS:

1 - "Please review your reference list to ensure that it is complete and correct. If you have cited papers that have been retracted, please include the rationale for doing so in the manuscript text, or remove these references and replace them with relevant current references. Any changes to the reference list should be mentioned in the rebuttal letter that accompanies your revised manuscript. If you need to cite a retracted article, indicate the article's retracted status in the References list and also include a citation and full reference for the retraction notice."

- **RESPONSE: 3 extra references were included to further discuss the results found as suggested. All other references were checked.**

2 - “Please amend your detailed Financial Disclosure statement. This is published with the article. It must therefore be completed in full sentences and contain the exact wording you wish to be published.”

- **<u>RESPONSE:</u> We have reviewed the details of the Financial Disclosure statement and have included that the funders had no role in study design, data collection and analysis, decision to publish, or preparation of the manuscript.**

3 - “We ask that a manuscript source file is provided at Revision. Please upload your manuscript file as a .doc, .docx, .rtf or .tex”

- **<u>RESPONSE:</u> We have included with the submission a manuscript version in .tex file format.**

4 - “The authors need to discuss about at what level of classification accuracy the NLP model can be used in practical situation to replace nurse on the emergency call. And also, the authors need to tone down the conclusion as 88.7% classification accuracy may not represent the different consequences of misclassified categories.”

- **<u>RESPONSE:</u> Thank you for your insightful suggestion. We have incorporated a detailed paragraph in the second to last section of the discussion, addressing the practical implications of the classification accuracy of our NLP model. We have emphasized that while the model offers significant efficacy, it is not intended to fully replace human judgment, especially given the potential consequences of misclassified categories. Instead, it seeks to complement and enhance the decision-making process of healthcare professionals during emergency calls. We believe this addition provides a balanced perspective on the utility and limitations of our model.**

REVIEWER COMMENTS:

1 - “The manuscript titled “AI-based approach for transcribing and classifying unstructured emergency call data: A methodological proposal” is original and attempts to leverage contributions that AI and ML is making in language translation and data

synthesis to support processing of emergency calls. To improve interest of wider community of health informatics researchers and clinicians in the rest of LMICs, I recommend that the authors include a distinctive discussion on what is happening in other LMICs and draw similarity/difference to state of practice in Brazil. This will support generalizability of the study findings."

- **<u>RESPONSE:</u> Thank you for your valuable suggestion to enhance the relevance of our study for a wider audience. In response to your recommendation, we have incorporated a dedicated paragraph in the second section of the discussion. This addition draws parallels between the challenges faced in Brazil and those in other LMICs, emphasizing the broader applicability and significance of our findings. By highlighting specific instances and challenges in LMICs, we aim to underscore the potential of our AI-based approach to address pressing needs not only in Brazil but also in similar contexts globally.**

2 - "The methodological processes presented is rigorous enough to for us to agree that the study findings are scientifically sound. However, we cannot be sure on the appropriateness of the data set created through artificial training corpus building. How did the authors address issues of written text viz a vie common practice of using informal expressions such as slang words in spoken language as would be the case in emergency situations?"

- **<u>RESPONSE:</u> Thank you for your comment. In response to your concerns regarding the appropriateness of the dataset created through artificial training corpus building, we have added a clarification in the "Artificial training corpus building" subsection under methods. We have emphasized that the generated examples encompass informality, slang words, and regionalisms consistent with the real transcriptions. This ensures that our dataset captures diverse forms of speech, including those informal expressions commonly found in emergency situations, thereby exposing the classifier to the variations intrinsic to the domain context. We believe this addition addresses the potential gap you highlighted and further strengthens the methodological rigor of our study.**

3 - “The results show an improvement in the word error rate for their proposed Ai-based model over the other models. However, the discussions did not reflect this as still being high to raise risks to patient life in case such an error in classification happened in highly critical emergency case. It would be appropriate to reflect on this in the writeup.”

- **RESPONSE:** **Thank you for your comments. In light of your comments, we have incorporated a comprehensive paragraph in the penultimate section of the discussion. This addition emphasizes the importance of professional validation, even with the improved accuracy of our AI-based model. We have highlighted the potential risks associated with relying solely on the model, especially in critical emergency situations, and underscored the value of human oversight in tandem with AI capabilities. By detailing scenarios such as calls from noisy environments or instances of truncated communication, we further illustrate the challenges and the necessity of a dual approach. Thus, rather than replacing human judgment, our proposal seeks to complement it, potentially enhancing healthcare professionals' efficiency and accuracy in critical situations.**

4 - “The conclusions are based on the finding of the study. And actually, the evidence presented in this work is substantial to warrant such conclusions. however, the lack of availability of the data set and or information about how it can be ethically accessed limits other researchers who might be interested in further exploring the domain.”

- **RESPONSE:** **Thank you for your comment. In response to your comment, we have updated the "Data Availability" section to clarify that the data used in this study contains sensitive participant information and sharing it openly would breach the guidelines set by SAMU in our data sharing agreement. However, to facilitate potential future research endeavors, we have also provided details on how interested researchers can contact the appropriate channels to potentially gain access to the data, ensuring ethical considerations are met.**

ADDITIONAL CHANGES: Beyond the requested revisions, upon reviewing the manuscript, we identified a few more details that warranted attention. We added acknowledgments in the "Acknowledgments" section to the research assistants who contributed to this study. We also updated the manuscript's abstract in the results section regarding the accuracy of the NLU model, which was previously stated as 88.7%. In fact, the NLU model's accuracy when tested on a validation subset was 73.9%, as described in the manuscript's results section.

CONCLUDING REMARKS: Again, thank you for giving us the opportunity to strengthen our manuscript with your valuable comments and queries. We have worked hard to incorporate your feedback and hope that these revisions persuade you to accept our submission.

Sincerely,

João Ricardo Nickenig Vissoci, PhD
Assistant professor of Emergency Medicine and Global Health
Director, Global Emergency Medicine Innovation and Implementation (GEMINI) Center
Duke University
Email: jnv4@duke.edu
